# Does individual pain threshold influence postoperative pain after connective tissue graft surgery?

**DOI:** 10.1007/s00784-026-07172-4

**Published:** 2026-09-25

**Authors:** Mariana Souza Calefi, Rafaela Stocker Salbego, Caique Andrade Santos, Rafael Sponchiado Cavallieri, Adriana Campos Passanezi Santana, Carla Andreotti Damante, Leonardo Rigoldi Bonjardim, Mariana Schutzer Ragghianti Zangrando

**Affiliations:** 1 Department of Prosthodontics and Periodontology, Bauru School of Dentistry, University of São Paulo, Bauru, Brazil; 2 Department of Biological Sciences, Bauru School of Dentistry, University of São Paulo, Bauru, Brazil

**Keywords:** Pain, Pain threshold, Periodontics, Autografts

## Abstract

**Objectives:**

Postoperative pain and discomfort remain common concerns in periodontal plastic surgery involving gingival grafts; however, the influence of individual pain sensitivity on postoperative outcomes remains poorly understood. This study evaluated whether preoperative pain sensitivity is associated with postoperative pain intensity and analgesic consumption after coronally advanced flap (CAF) combined with subepithelial connective tissue graft (SCTG).

**Materials and methods:**

In this exploratory clinical study, 25 participants undergoing CAF + SCTG were assessed preoperatively for psychological factors (pain catastrophizing and fear of pain) and cold pain sensitivity using a cold pressor test. Cold pain threshold (CPT) and cold pain tolerance (CPTol) were determined by recording the time to pain onset and maximum tolerated immersion in cold water, with corresponding visual analog scale (VAS, 0–100) scores. Postoperative pain, discomfort, and analgesic intake at donor and recipient sites were recorded daily for 14 days using VAS-based diaries. Hierarchical and K-means clustering identified postoperative pain profiles, and binomial logistic regression evaluated predictors of cluster membership.

**Results:**

Two postoperative pain profiles were identified. Cluster 1 showed lower pain intensity and lower analgesic consumption, whereas Cluster 2 exhibited higher postoperative pain and greater analgesic use. Higher initial pain intensity during the cold pressor test significantly increased the odds of belonging to Cluster 2, with each unit increase associated with an approximately 11.2% increase in the odds of membership. In contrast, cold pain threshold, pain tolerance, and psychological variables were not associated with postoperative pain profiles.

**Conclusions:**

Preoperative pain intensity during a cold pressor test may help identify patients at greater risk of postoperative pain after CAF + SCTG, suggesting a simple approach for preoperative risk stratification.

**Clinical relevance:**

Assessment of individual pain sensitivity before surgery may help clinicians anticipate postoperative discomfort and tailor analgesic strategies to improve patient-centered outcomes following root coverage procedures.

## Introduction

Periodontal plastic surgeries using subepithelial connective tissue grafts (SCTG) for the treatment of gingival recessions are considered the gold standard for root coverage procedures [1–5]. However, postoperative pain, morbidity, and occasional complications still represent a significant challenge, as they may affect personal and interpersonal daily activities such as eating, speaking, and socializing, with potential repercussions on patients’ quality of life [6–10].

Pain is a multidimensional experience, as it involves both sensory input and psychological processing in response to unpleasant sensations resulting from actual or potential tissue damage [11]. Several experimental methods have been developed to assess pain perception, individual pain thresholds, and pain tolerance levels [12]. The cold pressor test (CPTest), which involves the immersion of a limb—typically the hand—into ice-cold water, is widely used for this purpose [13]. The pain induced by this method is comparable to acute clinical pain, such as postoperative pain, which may last from a few minutes to several hours [13, 14]. Within this experimental context, two primary parameters are assessed: cold pain threshold (CPT) and cold pain tolerance (CPTol) [15].

The CPTest has demonstrated capacity to predict pain intensity and the development of prolonged pain following various surgical procedures [16–18]. Also, the tolerance could be measured, and some evidence suggests that patients with low pain tolerance to this test reported significantly higher pain scores at 2, 32, and 48 h after third molar surgery compared to those with high tolerance, as well as a significantly higher requirement for analgesic in the first 2 h following surgery [19]. Predictive models have also shown that sensory-discriminative and affective responses to the cold pressor stimulus are significant predictors of peak postoperative pain occurring approximately 3 h after surgery [17].

In addition to experimental pain tests, validated psychometric instruments are available to assess psychological conditions that may influence pain perception, such as the Pain Catastrophizing Scale (PCS) and the Fear of Pain Questionnaire–III (FPQ-III). Pain catastrophizing refers to an exaggerated and negative orientation toward noxious stimuli and has been associated with greater pain intensity in experimental models. Although this scale is primarily designed for chronic pain conditions, it may also reveal an unfavorable psychological profile even in acute pain contexts. In orthognathic surgery, higher PCS magnification predicted greater acute postoperative pain, with a multivariable model explaining substantial variance in pain burden [20]. In third-molar surgery, analgesic use also tended to be higher in patients with high catastrophizing [21]. In an experimental incisor pain model, unpredictable pain stimuli increased both pain intensity and anxiety, and participants with higher PCS scores exhibited greater increases in pain under these conditions [22].

Similarly, fear of pain assessed by the FPQ-III has been shown to predict individual responses to experimentally induced pain [23]. Fear of pain appears to operate through both expectation and memory, as patients with greater fear of pain predict and later recall more intense pain and anxiety than less fearful patients. Moreover, recalled anxiety strongly contributes to recalled dental pain after extraction [24]. Also, anticipated pain also tracks anxiety across procedures, and pain and bleeding were the most common anxiety triggers [25].

To the best of our knowledge, there are no reports in the literature evaluating the impact of pre-treatment individual pain perception and psychosocial factors on postoperative pain following gingival graft surgeries. Therefore, the aim of the present study was to evaluate whether values obtained from experimentally determined individual pain thresholds and pain tolerance (pain intensity and time) could predict the likelihood of greater postoperative pain and the need for rescue analgesics in patients undergoing root coverage surgery with SCTG associated with a coronally advanced flap (CAF). The secondary objective was to evaluate the influence of psychological variables, assessed through questionnaires, on postoperative pain intensity and the use of rescue analgesics.

## Materials and methods

The experimental protocol was submitted and approved by the Human Research Ethics Committee of the Bauru School of Dentistry, University of São Paulo (FOB-USP) (CAAE: 19692019.1.0000.5417). All participants provided written informed consent prior to participation. The clinical study was registered in the Brazilian Registry of Clinical Trials (ReBEC) (RBR-93ccq38). Twenty-five individuals were selected for treatment at the Department of Periodontology of the Bauru School of Dentistry, University of São Paulo (FOB-USP). The inclusion criteria were systemically healthy individuals aged between 18 and 70 years, normal and healthy palatal area, and a clinical diagnosis of bilateral multiple gingival recessions classified as RT1 or RT2, with at least one gingival recession ≥ 2 mm, involving canines and premolars. The exclusion criteria included: the presence of prostheses with palatal coverage, previous graft harvesting from the palatal donor area, teeth with mobility, smokers, pregnant or lactating women, history of periodontal disease or recurrent abscess formation, use of medications (anticonvulsants, antihypertensives, contraceptives, or immunosuppressive agents) or drugs that could interfere with wound healing, patients with any history or clinical/laboratory evidence of hepatic impairment, hypersensitivity to nimesulide or dexamethasone and poor oral hygiene levels (plaque index and bleeding index > 20%).

All selected patients underwent initial periodontal therapy. This therapeutic phase aimed to control local factors that could negatively influence the clinical condition prior to the surgical procedure. The treatment proposed for root coverage of gingival recessions consisted of the association of SCTG plus CAF [26]. The flap in the recipient area was prepared using a split–full–split thickness design and stabilized approximately 2 mm coronal to the cementoenamel junction (CEJ) [27]. The SCTG was inserted and stabilized at the level of the CEJ and was harvested from the palatal donor site using the de-epithelialization technique [28]. The grafts were standardized with a mesiodistal extension corresponding to two teeth (canine/premolar or two premolars), a height of 7 mm, and a thickness of 1.5 mm. Monofilament nylon 5 − 0 sutures (Techsuture, SP-Brazil) were used in both the recipient and donor areas and were removed after 14 days. Postoperative care was also standardized and included oral preoperative medication (8 mg dexamethasone one hour before the surgical procedure) and postoperative medication (200 mg nimesulide twice daily on the first and second days and 100 mg twice daily on the third and fourth days). Participants were instructed to take 750 mg paracetamol every 6 h as rescue medication in case of pain. In addition, mouth rinsing with 0.12% chlorhexidine twice daily for 14 days was prescribed. Participants were instructed not to use dental floss or a toothbrush in the recipient area during this period.

### Postoperative pain and discomfort assessment

Patients completed a postoperative diary [29] during the first 14 days after surgery, recording pain and discomfort using visual analog scales (VAS), where 0 indicated no pain and 100 indicated the worst pain imaginable. Pain was recorded daily for both the recipient and donor sites of SCTG, according to the instructions previously provided to the participants. The need for rescue medication and the number of analgesic consumed were also recorded daily by the patients.

### Cold pain threshold (CPT) and cold pain tolerance (CPTol)

**Fig. 1:**
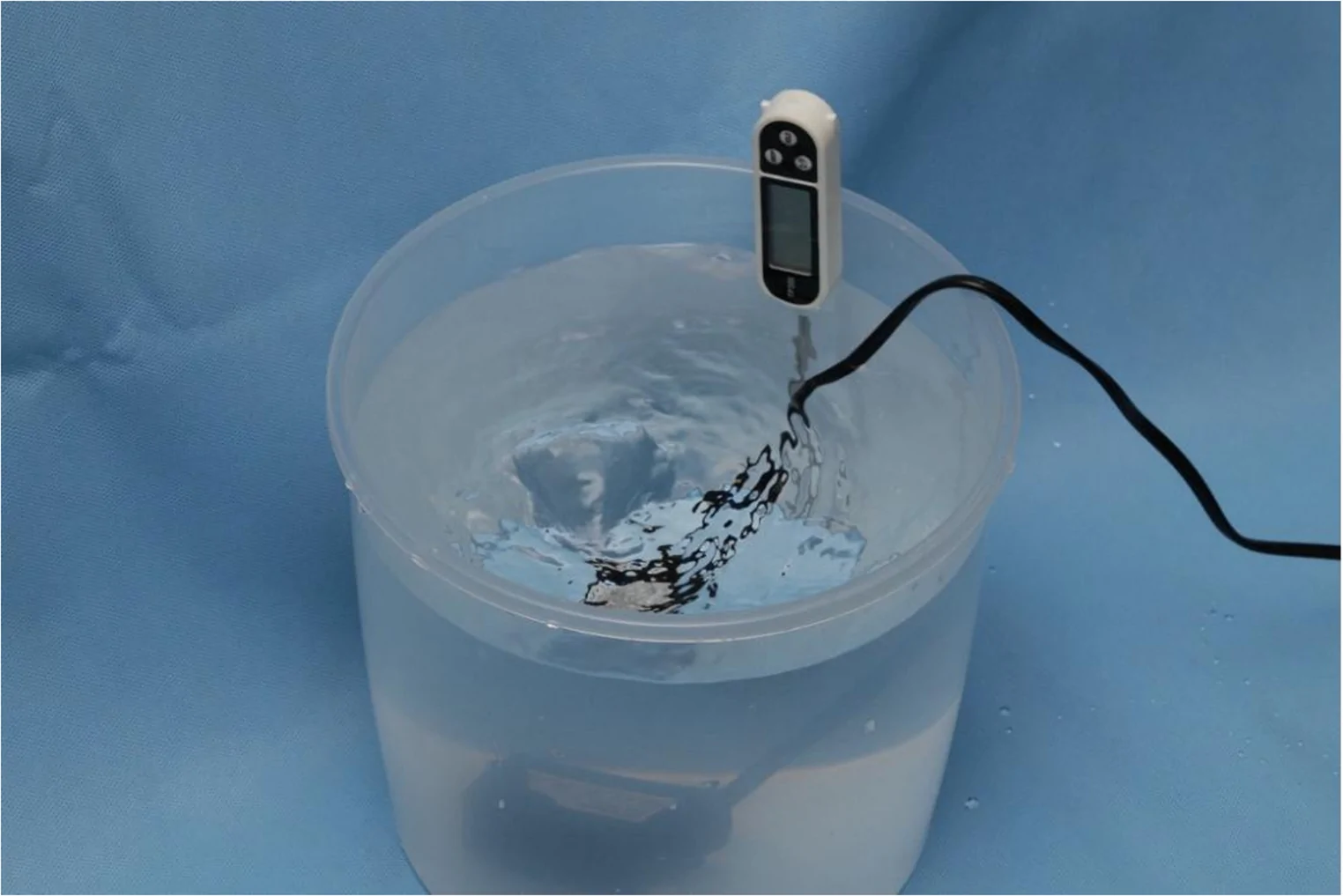
Illustrative image of the water circulation system and temperature control on cold pressor test

**Fig. 2:**
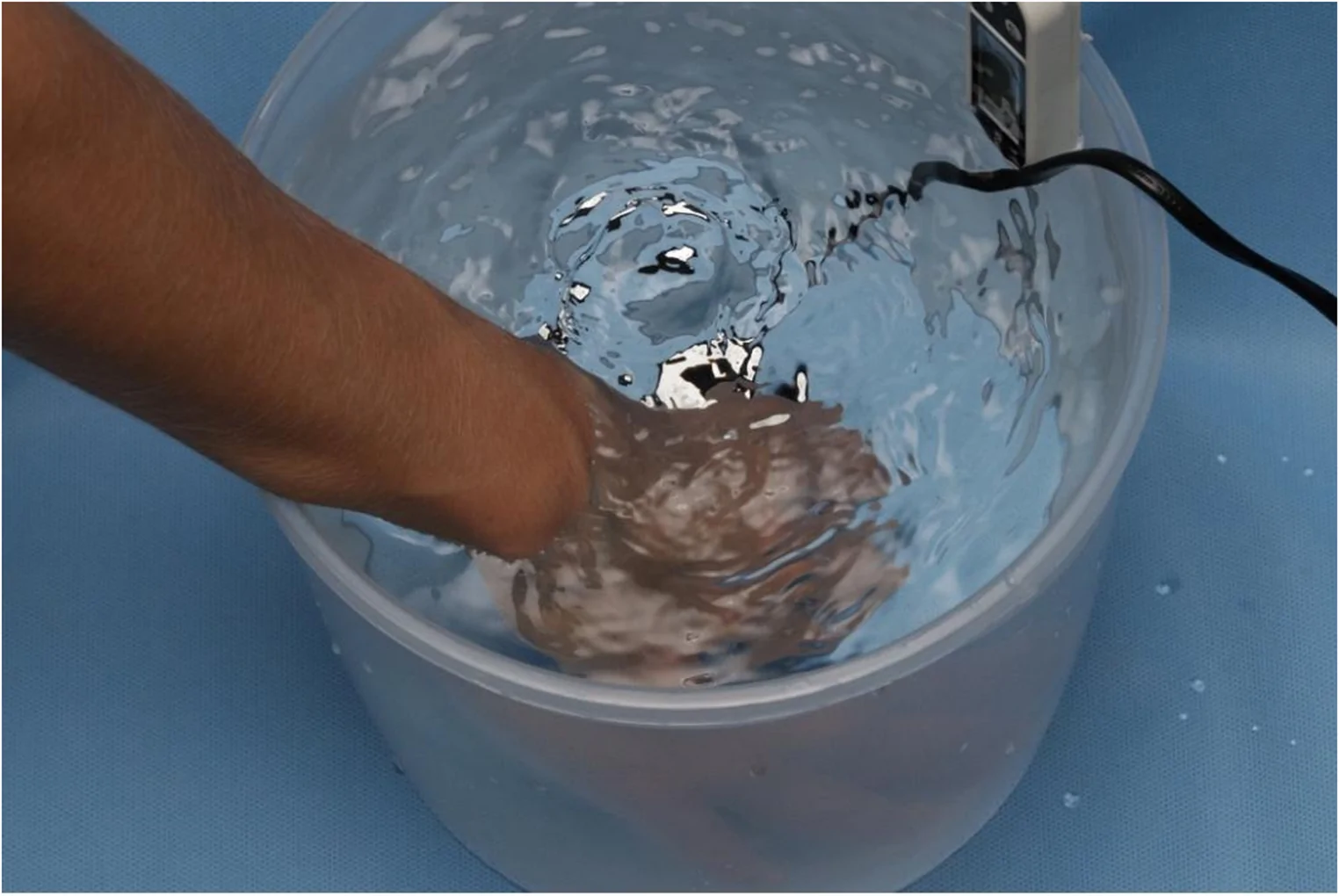
Simulation of the hand position to be maintained during the cold pressor test

The CPT and CPTol tests [30, 31] were performed during the preoperative appointment, and consisted of immersing the open and immobile non-dominant hand, up to five centimeters above the wrist, in a container filled with water maintained at a constant temperature of 5 °C. The water temperature was maintained using a submerged pump (Dttra, flow rate: 200 L/h) and monitored with a digital thermometer (Uny Home). The standardized temperature of 5 °C was considered sufficient to induce pain in the study population; however, the intensity was tolerable for a short period of time (ranging from a few seconds to a few minutes) (Figs. 1 and 2).

Prior to the tests, participants were instructed by the same examiner (MSC) to report when the sensation in the hand changed from being merely cold to becoming painful (pain threshold) and to remove their hand from the container when the painful sensation became intolerable (pain tolerance). During the tests, immersion times (in seconds) and pain intensity (VAS) were recorded.

Participants immersed their hand in the container with water maintained at a controlled temperature of 5 °C, and two digital timers were immediately activated. One timer recorded the cold pain threshold time, defined as the moment when the participant reported that the sensation of cold water had become slightly painful. The participant then continued to keep the hand immersed for the evaluation of pain tolerance, and the time on the second timer was recorded when the painful sensation became intolerable, prompting the participant to voluntarily withdraw the hand.

In both tests, participants were asked to report the intensity of their pain using a visual analog scale (VAS), in which 0 corresponded to no pain and 100 to the worst pain imaginable. Pain intensity was recorded at two time points: one corresponding to the pain threshold and the other to pain tolerance.

The maximum duration of the test was set at 3 min, at which point participants would be asked to remove their hand from the water. Participants were not informed of this maximum limit to avoid influencing the test results.

Thus, the following measures were recorded:

1. CPT- Cold pain threshold (time in seconds from hand immersion in the water until the onset of painful sensation).
2. CPtol- Cold pain tolerance (time in seconds from hand immersion in the water until the participant voluntarily withdrew the hand).
3. Pain intensity at the cold pain threshold (VAS CPT).
4. Pain intensity at cold pain tolerance (VAS CPtol).

### Psychosocial questionnaires

Two adapted and validated questionnaires were used: the Pain Catastrophizing Scale (PCS) [32] e and the Fear of Pain Questionnaire (FPQ-III) [33]. The questionnaires were administered prior to exposure to the experimental pain stimuli and during appointments preceding the surgical procedure, to avoid any potential influence of fear or stress on the participants’ responses. All participants received standardized instructions from the same examiner (MSC).

The PCS consists of 13 items, which were rated by participants on a scale ranging from 0 (never) to 4 (all the time). The questionnaire comprises three domains: rumination, magnification, and helplessness. The analysis was based on the sum of the total scores across all domains, as previously described and validated. The total PCS score may range from 0 to 52, with higher scores indicating a greater degree of pain catastrophizing. Participants received standardized instructions regarding the Questionnaire.

The FPQ-III consists of 30 items, which were also rated by participants on a 5-point scale ranging from 1 (not at all) to 5 (extreme). The questionnaire includes three subscales: minor pain, moderate pain, and severe pain. Higher total scores (ranging from 30 to 150) indicate greater levels of fear of pain. Participants received standardized instructions regarding the questionnaires.

### Statistical analysis

Data were tested for normality using Shapiro–Wilk test (*p* > 0.05). Descriptive analyses of data that followed a normal distribution were presented as mean (standard deviation), whereas data that did not follow a normal distribution were presented as median (interquartile range). Differences between gender were analyzed using MANOVA. Initially, Hierarchical Clustering was performed to identify the optimal number of subgroups in the dataset and to map initial patterns of similarity. This hierarchical method provides an initial overview, visualized through dendrograms, and is useful for exploring patterns in the data without requiring a predefined number of groups.

Subsequently, K-means Clustering was used to refine cluster partitioning and to explore patterns based on the pain diaries from both the graft recipient and donor sites, as well as the total number of analgesics consumed during the 14-day postoperative period. Finally, to identify significant predictors influencing the likelihood of individuals belonging to one of the identified clusters, Binomial Logistic Regression analysis was performed.

## Results

The total sample comprised 25 participants with a mean age of 42.8 (± 8.35) years. Data collection was performed during a preoperative appointment and included evaluation of CPT (time in seconds from the beginning of the test until the initial report of pain) and CPTol (maximum tolerated time in seconds), along with their respective pain intensity scores (VAS). A percentage variation analysis (relative VAS) was performed to evaluate the proportional difference between the initial and final pain levels and for PCS and FPQ-III (Table 1).

**Table 1:** General description of the study population and preoperative variables. *CPT* cold pain threshold, *CPTol* cold pain tolerance, *VAS* visual analogue scale, *PCS* pain catastrophizing scale, *FPQ-III* fear of pain questionnaire

Variable	Median (25º–75º)
CPT	13,8 (9,83–21,4)
CPTol	28,6 (22,8–52,8)
VAS – CPT	60,0 (50,0–80,0)
Relative VAS	40,0 (25,0–74,5)
VAS – CPTol	95,0 (80,0–100)
PCS (0–52)	22,5 (15,0–33,0)
FPQ-III (30–150)	82,0 (63,8–92,5)

The means and variability were analyzed separately for women and men (Table 2). The mean age of women was 44.5 (± 6.29) years, whereas the mean age of men was 38.4 (± 12) years. Women demonstrated a lower pain threshold and greater pain intensity at the time of the pain threshold test (VAS–CPT), while men showed greater variability in pain tolerance (higher standard deviation).

**Table 2:** – Gender-based statistics of preoperative variables. *CPT* cold pain threshold, *CPTol* cold pain tolerance, *VAS* visual analogue scale

Variable	Female (mean ± sd)	Male (mean ± sd)	*P*-value
CPT	12.7 ± 4.2	30.3 ± 15.7	< 0.001
CPTol	29.8 ± 12.4	93.5 ± 70.0	0.003
VAS – CPT	66.4 ± 16.9	55.0 ± 18.7	0.196
Relative VAS	51.1 ± 36.4	45.7 ± 29.7	0.753
VAS – CPTol	95.0 ± 7.6	76.7 ± 13.7	0.001

### Cluster-based statistics

Data related to postoperative pain (recipient and donor sites), reported by the patients (VAS), and analgesic consumption were extracted from the 14-day postoperative diary. These data were subjected to Hierarchical Clustering, which produced a dendrogram with a visual pattern suggestive of two main groups of individuals. Based on these findings, K-means clustering was subsequently performed with the number of clusters pre-specified as two.

The clusters were formed based on the following variables: pain intensity in both graft recipient and donor sites, and the total number of analgesics consumed during the 14-day postoperative period. This analysis resulted in two clusters: Cluster 1 (characterized by centroid values indicating lower pain levels and lower analgesic consumption) and Cluster 2 (characterized by higher centroid values for pain and analgesic consumption) **(**Table 3**)**.

**Table 3:** Description of the centroid values for each clusters. *VAS* visual analogue scale

	Cluster 1	Cluster 2
VAS – Donor Site	15.00 (10.00–27.31)	61.43 (33.21–68.93)
VAS – Recipient Site	8.08 (6.15–9.04)	44.64 (15.71–51.43)
Analgesic Consumption	0.42 (0.31–0.71)	1.21 (0.29–1.39)

**Fig. 3:**
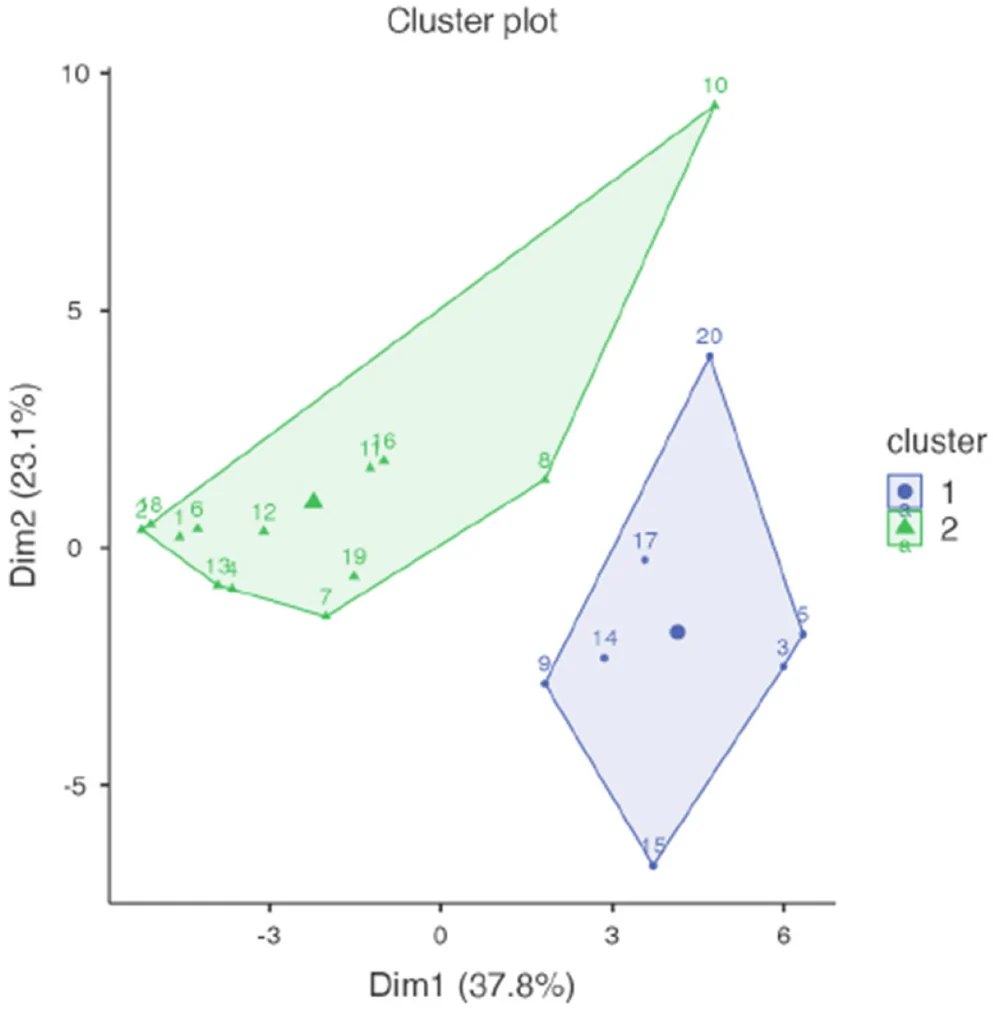
K-means clustering. The graph demonstrates a clear stabilization point at two clusters, confirming this choice as optimal for the analyzed data

The centroid analysis confirmed significant differences between the groups. The validation of the optimal number of clusters was performed using the Elbow Method. The graph used to identify the optimal number of clusters was generated based on the Within-Cluster Sum of Squares (WSS) **(**Fig. 3**)** for different numbers of clusters, as calculated by the K-means algorithm. WSS values were computed for different cluster configurations and plotted in a graph following the Elbow method approach, which identifies the “elbow” point where the reduction in WSS begins to stabilize, indicating the optimal number of clusters. This analysis confirmed that two clusters represented the optimal solution.

The analysis of relative VAS (percentage variation analysis) allowed the evaluation of how patients’ pain varied between the groups. In Cluster 1, the median value was 50%, whereas in Cluster 2 the median was 25%. Based on these percentage variations, it can be inferred that patients in Cluster 1 exhibited a greater proportional change in pain intensity between threshold and tolerance. The marked difference in relative VAS between the clusters suggests that patients belonging to Cluster 2 may be less capable of tolerating progressive increases in pain intensity. The median pain catastrophizing score was higher in Cluster 2, whereas fear of pain scores were higher in Cluster 1, possibly indicating the presence of distinct psychological subgroups.

### Binomial logistic regression

Binomial logistic regression was performed to evaluate which factors influenced the likelihood of individuals belonging to Cluster 1 or Cluster 2 (Table 4). The initial model structure included the following predictor variables: sex, age, fear of pain, pain catastrophizing, threshold (s), tolerance (s), VAS threshold, VAS tolerance, and relative VAS. As several variables represented similar outcomes, high multicollinearity was observed. Therefore, variables with the highest variance inflation factor (VIF) values were sequentially removed until all remaining variables were within the acceptable threshold (VIF < 2). Cluster membership (Cluster 1 or Cluster 2) was defined as the binomial outcome variable. Thus, the likelihood of belonging to Cluster 2 compared with Cluster 1 was modeled using the following predictor variables: fear of pain, sex, and VAS threshold. After verification of the model assumptions, the Cox and Snell R² was 0.440, indicating that approximately 44.0% of the variance in cluster membership was explained by the variables included in the model. The global model test showed deviance = 18.2 and AIC = 26.2, with overall statistical significance (χ² = 7.42; df = 2; *p* = 0.024), confirming that the model provided a good fit to the data and that at least one of the predictor variables was significantly associated with belonging to Cluster 2.

**Table 4:** Binomial logistic regression analysis. *CPT* cold pain threshold, *CPTol* cold pain tolerance, *VAS* visual analogue scale, *FPQ-III* fear of pain questionnaire, *VIF* variance inflation factor

Predictor	Estimate (β)	Standard error	Z	*P*-value	Or (e^β)	95% CI lower	95% CI upper	VIF	CPTol
Intercept	−5.2102	3.9123	−1.332	0.183	0.0055	0.0000	11.6800	-	-
FPQ-III	−0.0338	0.0410	−0.822	0.411	0.9668	0.8921	1.0500	1.55	0.643
Sex (m–f)	0.8210	1.6054	0.511	0.609	2.2727	0.0977	52.8500	1.55	0.644
VAS – CPT	0.1062	0.0505	2.104	0.035	1.1120	1.0073	1.2300	1.75	0.573

## Discussion

The present study investigated the potential effects of individual pain threshold and tolerance to cold stimulation (time and intensity), as well as psychological factors, on the likelihood of belonging to a group presenting greater postoperative pain intensity and higher analgesic consumption following root coverage surgery with SCTG plus CAF. Two main clusters were identified. Cluster 1 comprised individuals with lower postoperative pain intensity and lower analgesic consumption, whereas Cluster 2 included individuals with higher postoperative pain intensity and greater analgesic consumption. These findings indicate that, even with standardized surgical procedures, pharmacological protocols, and postoperative care, a subset of patients may still be at risk of insufficient postoperative analgesia. In addition, the measurement of cold pain intensity in the preoperative period was able to predict postoperative pain intensity. In the present study, the variable pain intensity at the pain threshold (VAS CPT) was found to influence the likelihood of a patient belonging to Cluster 2 (greater postoperative pain intensity and higher analgesic consumption). Each unit increase in pain intensity (VAS CPT) increased this likelihood by approximately 11.20%. This pain assessment has been shown to be a reliable quantitative sensory testing measure [15] and has been widely used in experimental and clinical pain studies [11, 14]. Furthermore, cold stimulation is easily applied and is typically well accepted by patients.

These findings are consistent with previous evidence [16–20, 34–36] suggesting that baseline pain sensitivity is a strong predictor of postoperative pain. Patients with greater baseline pain sensitivity tend to experience more intense postoperative pain following surgical procedures and require greater analgesic consumption. This phenomenon may be related to both physiological mechanisms and cognitive-affective factors that modulate pain perception and may amplify it [16–20, 34–36]. This association is further reinforced by recent systematic evidence mapping the landscape of preoperative risk factors for acute postoperative outcomes [37]. Although current literature highlights demographic and psychometric indicators, the present study refines this paradigm by showing that objective, baseline sensory responses provide critical predictive insights that may complement traditional psychological questionnaires in predicting patient-centered morbidity after gingival grafts. Corroborating this perspective, clinical necessity of identifying robust preoperative predictors of acute postsurgical pain is essential to tailor individualized management strategies [37]. Our findings add new evidence supporting the use of an easily applicable experimental model to stratify pain profiles in periodontal plastic surgery.

Differences between gender were also observed. Men and women differed in variables related to pain threshold time, pain tolerance time, and pain intensity. Men demonstrated higher pain thresholds, greater tolerance, and lower pain scores during the cold stimulation tests. This result is consistent with findings in the literature, which provide substantial evidence that women generally exhibit lower pain thresholds [32, 38, 39]. However, the variables that influenced cluster allocation (such as initial VAS and relative VAS) did not show significant differences between gender. Therefore, although general differences between men and women exist, these differences do not appear to directly influence cluster dynamics. Although men and women differ in pain sensitivity [31, 39], these differences did not directly affect the key variables influencing the model.

It is well established in the literature that preoperative anxiety levels influence pain perception in periodontal, implant and third molar surgeries, representing an important implication for clinical practice [34, 35, 40, 41]. The psychological factors evaluated with PCS and FPQ-III differed between clusters: pain catastrophizing was higher in Cluster 2, which was associated with higher postoperative pain levels and greater analgesic consumption, whereas fear of pain scores were higher in Cluster 1. It is possible that catastrophizing, which is a psychological profile often associated with lower pain thresholds and frequently observed in patients with chronic pain, may also reflect an unfavorable profile for tolerating acute postoperative pain. Pain is a subjective and multidimensional experience that is often not adequately managed in clinical practice. Although previous studies have investigated predictors of postoperative pain across various surgical and dental procedures [16, 17, 19–21, 23, 42], none has specifically examined these factors in the context of periodontal plastic surgery. Moreover, the evaluation of psychological and behavioral factors is often neglected in the management of postoperative pain.

Despite the statistically significant findings, the results should be interpreted with caution due to the exploratory nature of this study, the relatively small sample size and limited statistical power. Logistic regression models developed from limited datasets may be more susceptible to unstable parameter estimates, wider confidence intervals, and potential overfitting, which can affect the generalizability. Therefore, although pain intensity at the cold pain threshold emerged as a significant predictor of postoperative pain profile, these results should be considered preliminary and hypothesis-generating. Future studies with a priori sample size calculations and larger, more diverse samples are needed to confirm these outcomes and establish their clinical applicability.

Thus, the study presented innovative information about individual profile of pain threshold and tolerance associated with root coverage procedures. Individuals presenting lower CPT and CPtol values (measured in seconds), combined with higher VAS pain for both and, smaller variation between them, may have a greater tendency to experience more intense pain. Therefore, a simple and rapid preoperative test (CPT and CPTol) may be useful in identifying patients who are more likely to experience greater pain following SCTG plus CAF. Preoperative assessment may help predict which patients are more susceptible to experiencing higher postoperative pain and increased analgesic consumption. This test could assist healthcare professionals in adapting treatment protocols to the specific needs of each patient, thereby improving postoperative outcomes and patient satisfaction. Accordingly, the importance of establishing individualized preoperative strategies for treatment and postoperative care aimed at controlling pain-related outcomes should be emphasized. The early identification of predictors of postoperative pain risk allows for more effective interventions and promotes patient-centered care.

## Conclusion

The findings of this study indicated that individual variability in pain sensitivity contributes to differences in postoperative pain and analgesic consumption after root coverage procedures with SCTG plus CAF. Even under standardized surgical and pharmacological protocols, a subgroup of patients exhibited a higher risk of postoperative pain. Preoperative assessment of pain intensity to cold stimulation emerged as a relevant predictor of postoperative pain experience, supporting its potential use in identifying patients who may benefit from individualized pain management strategies.

## Data Availability

The datasets generated and/or analyzed during the current study are not publicly available due to privacy and ethical restrictions but are available on reasonable request.
